# Case report: A colorectal cancer patient with microsatellite instability-high and *MSH2* germline mutation failed to respond to anti-PD-1 immunotherapy

**DOI:** 10.3389/fimmu.2022.953421

**Published:** 2022-08-05

**Authors:** Qun Zhang, Jing Hu, Yaping Zhang, Li Li, Ting Wang, Xiaoping Qian

**Affiliations:** ^1^ Comprehensive Cancer Centre of Nanjing Drum Tower Hospital, Medical School of Nanjing University, Clinical Cancer Institute of Nanjing University, Nanjing, China; ^2^ The Comprehensive Cancer Center, Nanjing Drum Tower Hospital Clinical College of Xuzhou Medical University, Nanjing, China; ^3^ Department of Pathology, Nanjing University Medical School Affiliated Nanjing Drum Tower Hospital, Nanjing, China

**Keywords:** microsatellite instability-high, colorectal cancer, anti-PD1 therapy, lynch syndrome, resistance

## Abstract

Lynch syndrome (LS) is characterized by germline mutations in the DNA mismatch repair (MMR) genes. In colorectal cancer (CRC), germline mutations of DNA MMR genes commonly lead to microsatellite instability-high (MSI-H) subtype formation. Recent studies have demonstrated that CRC patients with MSI-H or mismatch repair-deficient (dMMR) status can benefit from anti-PD1 immunotherapy. However, almost 50% of CRC patients with MSI-H status do not respond to it. It is reported that heterogeneity of tumor and abnormal activation of cancer-related signaling pathways contribute to resistance to anti-PD1 therapy. To improve the clinical efficacy of such patients, the underlying mechanisms of resistance to anti-PD1 treatment must be explored. In this case, we describe an LS-associated CRC patient with MSI-H who suffered resistance to anti-PD1 therapy. Here, we attempted to elucidate the potential reasons, and thus appropriate strategies may be derived to overcome this clinical problem.

## Introduction

Lynch syndrome (LS) is caused by germline inactivation of one allele of genes involved in the mismatch repair (MMR) system, namely MLH1, MSH2, MSH6, and PMS2 (1). Inactivation of MLH1 or PMS2 alleles is the most frequent and is associated with approximately 80% of LS cases (2). LS-associated CRC usually presents as a microsatellite instability-high (MSI-H) subtype. Several clinical trials have confirmed CRC patients with MSI-H/dMMR are the beneficiaries of anti-PD1 therapy, and the overall response rate varies from 40% to 60%. Based on these data, anti-PD1 monoclonal antibody (mAb) was approved for first-line treatment of advanced MSI-H CRC, which revolutionized the treatment mode of metastatic colorectal cancer (mCRC) (3). Clinical data have demonstrated the long-lasting and stable antitumor efficacy of anti-PD1 therapy in MSI-H mCRC (4).

Despite the remarkable efficacy of anti-PD1 therapy in advanced MSI-H/dMMR CRC patients, nearly half of MSI-H/dMMR CRC patients do not respond to it. Studies have indicated that the underlying resistance mechanisms and the most straightforward reason are the absence of tumor antigens leads to a lack of recognition by T cells (5). Recently, abnormal activation of multiple tumor-associated signaling has been identified to contribute to the resistance of anti-PD1 mAb. The mitogen-activated protein kinase (MAPK) pathway, the PTEN expression, the PI3K signaling, and the WNT/b-catenin signaling pathway are the main immune­evasive oncogenic signaling pathways. Moreover, loss of interferon-gamma signaling pathways and lack of tumor antigen expression were also involved in tumor immune escape (6–11). With the development of gene sequencing platforms, sequencing analysis of MSI-H/dMMR subtypes of CRC revealed that some specific gene mutations also lead to resistance to PD1 mab therapy. Here, we present a CRC patient with MSI-H and germline MSH2 mutation who failed to respond to anti-PD1 treatment. Immunohistochemical (IHC), PCR, and next-generation (NGS) sequencing assays were used to explore the underlying mechanism of this resistance.

## Case description

### Case presentation and treatment

**Figure 1:**
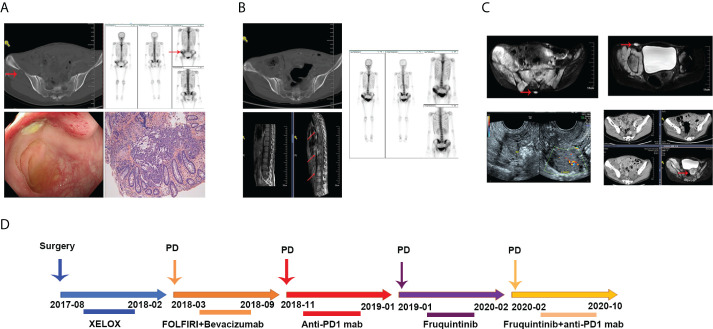
**(A)** Radiologic images of tumor metastases after the XEOLX therapy. **(B)** Radiologic images of tumor metastases after the second-line treatment. **(C)** Radiologic images of tumor metastases after anti-PD1 immunotherapy. **(D)** The entire treatment process of this patient.

A 27-year-old young female patient presented with abdominal pain and with no excrement for 1day and was sent to the hospital for physical examination in July 2017. Colonoscopy examination revealed a space-occupying lesion 30cm from the anal margin. Pathological examination suggested poorly differentiated adenocarcinoma. Computed Tomography (CT) examination showed no metastasis. Then, this patient underwent a radical resection of left colon cancer. The postoperative pathological stage was pT3N2aM0. Postoperative baseline assessment showed no recurrence and metastasis in this patient. We chose the XELOX regimen as postoperative adjuvant chemotherapy. However, this patient had progressive disease (PD) after six cycles of XELOX chemotherapy. ECT and CT examination suggested bone destruction of the right iliac crest, which was considered metastasis. Colonoscopy revealed a neoplasm at the top of the anastomosis, and pathology revealed a moderately differentiated adenocarcinoma (**Figure 1A**). Since genetic test results suggested a *KRAS* gene mutation in this patient, bevacizumab combined with the FOLFIRI regimen was used as a second-line treatment for her. In addition, we performed local radiotherapy on her right iliac crest metastatic lesion. However, after two cycles of combined targeted therapy with chemotherapy treatment, she developed disease progression again, presenting with enlarged tissue mass shadow around the right iliac crest and multiple metastases in the thoracolumbosacral vertebral body and appendages (**Figure 1B**). We reviewed her tumor tissue immunohistochemical (IHC) results and found a loss of MSH2 protein (**Figure 3B**). PCR and NGS tests further confirmed an MSI-H status in this patient. NGS tests also indicated a high tumor mutation burden (TMB) in the patient’s peripheral blood and tumor tissue, suggesting that the patient is likely to benefit from PD1 mab treatment. According to it, we chose anti-PD1 therapy (Tislelizumab 200mg every three weeks) as the third-line treatment. Unfortunately, this patient experienced a rapid progression again with widespread metastases including bone, ovary, and retroperitoneal lymph nodes after three cycles of anti-PD1 therapy (**Figure 1C**). When that, we chose furoquitinib and furoquitinib combined with sindilizumab as the follow-up treatment for her. During this period, this patient’s lesions were in a state of slow progression. Due to her poor physical condition, she discontinued therapy in October 2020 and received the best supportive care. She died in April 2021. To intuitively express this patient’s treatment process and efficacy, we listed the entire treatment process in **Figure 1D**.

### Family history

**Figure 2:**
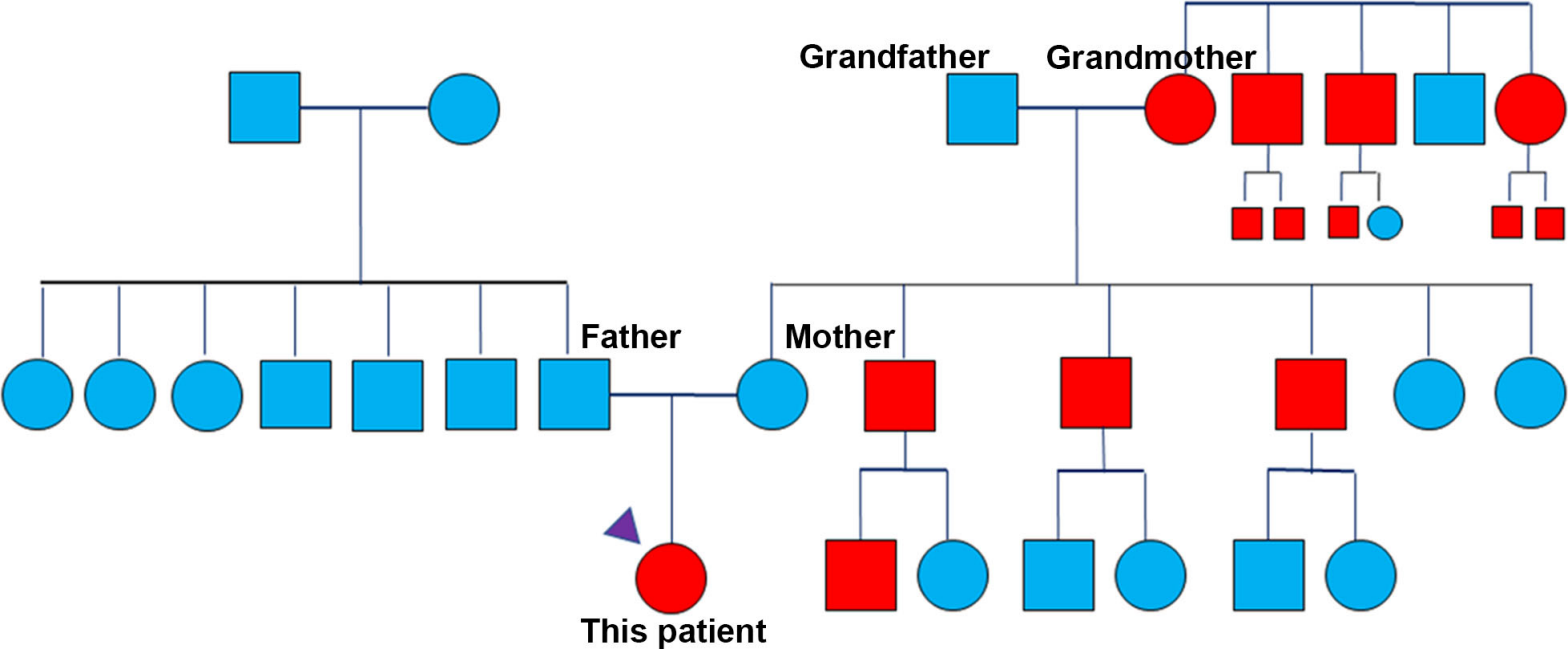
The characteristics of her family history.

Interestingly, when we reviewed her family history, it was worth noting that her grandmother had CRC, three uncles had CRC, and her grandmother’s siblings and their children all had CRC. Her family history fulfilled Amsterdam criteria. We presented her family history in **Figure 2**. NGS tests on blood and tumor tissue showed this patient had an *MSH2* germline mutation. Based on the above clinical and laboratory findings, she was diagnosed as a Lynch syndrome (LS) associated MSI-H CRC patient.

### IHC test and gene analysis

**Figure 3:**
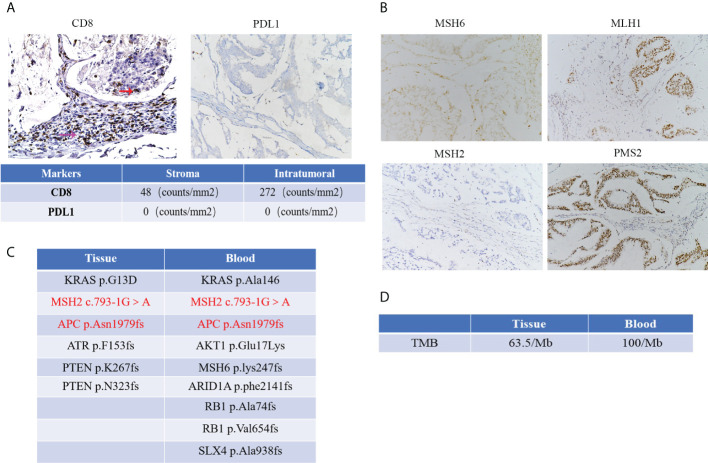
**(A)** The protein expression of CD8 and PDL1 and red arrow represents CD8 positive T cells in the tumor, and the purple arrow represents CD8 positive T cells in the stroma. Quantification results of CD8 and PDL1 expression by IHC with the tumor and tumor stroma. (Microscopic magnification×200) **(B)** IHC images of MMR protein expression in this patient (Microscopic magnification×200); **(C)** NGS detection showed several gene mutations with high frequency from peripheral blood and tumor tissue in this patient. **(D)** Results of TMB detection from peripheral blood and tumor tissue in this patient.

We used IHC assay to detect the protein expression of MLH1, MSH2, MSH6, PMS2, CD8, and PD-L1 in the formalin-fixed, paraffin-embedded (FFPE) tumor tissues. For quantitative analysis, we used ipp software to analyze the density of CD8 and PDL1. These data showed a small number of CD8+T cells were infiltrated in the tumor, and a large number of CD8+T cells were infiltrated in the stroma (**Figure 3A**). And we found a negative protein expression of PDL1 in this patient (**Figure 3A**).

The tissue DNA was extracted with a QIAamp DNA FFPE tissue kit (Qiagen, Valencia, CA, USA). Circulating tumor DNA (ctDNA) was extracted from plasma using the QIAamp Circulating Nucleic Acid Kit (Qiagen, Valencia, CA, USA). The tissue DNA and ctDNA were measured by Qubit 2.0 Fluorometer with a Qubit double-stranded DNA assay kit (Life Technologies, Carlsbad, CA, USA). Capture-based targeted sequencing was performed on tumor tissue and plasma samples using a panel (OncoScreen, Burning Rock Biotech, Guangzhou, China) consisting of 520 cancer-related genes. Sequencing data analysis was performed by OncoScreen Plus™. The result from the peripheral blood test revealed nine gene mutations with high frequency, including *KRAS* p.Ala146, *MSH2* c.793-1G>A, *MSH6* p.lys247fs, *AKT1* p.Glu17Lys, *APC* p.Asn1979fs, *ARID1A* p.phe2141fs, *RB1* p.Ala74fs, *RB1* p.Val654fs and *SLX4* p.Ala938fs. The result from FFPE showed six gene mutations with high frequency, including *KRAS* p.G13D, *MSH2* c.793-1G>A, *APC* p.Asn1979fs, *ATR* p.F153fs, *PTEN* p.K267fs and *PTEN* p.N323fs. These mutations were showed in **Figure 3C**. In addition, the results of tumor mutation burden from peripheral blood and tumor tissue were 100/Mb and 63.5/Mb, respectively (**Figure 3D**).

## Discussion

This is a case of failure from anti-PD1 therapy in LS-associated MSI-H CRC. This is also a CRC case to describe characteristics such as TMB, tumor immune infiltration factors, and some gene alterations in LS-associated MSI-H CRC patients. In this case, we tried to elucidate the reasons for resistance to ant-PD1 immunotherapy.

Two forms of testing are commonly used in screening MMR or MSI status. IHC was used for detecting MMR proteins and PCR testing for MSI. Due to the development of gene sequencing platforms, NGS has been applied more and more in gene detection. In this case, the IHC assay revealed a loss of MSH2 protein expression. Besides, the PCR test also showed all five single nucleotide sites (BAT-25, MONO-27, CAT-25, BAT-26, and NR-24) were changed. Thus, there is no doubt that this patient is an MSI-H/dMMR CRC patient. As a germline mutation, LS-associated tumors are commonly microsatellite unstable. In this case, the NGS test showed this patient had an MSH2 germline mutation and high TMB. Combined with her family history, she was diagnosed with an LS-associated MSI-H CRC.

Recent studies have demonstrated that solid tumors with MSI-H/dMMR subset commonly obtained a favorable response from anti-PD1 mAb, including CRC (12–14). The excellent efficacy of anti-PD1 therapy in treating MSI-H/dMMR CRCs is associated with the high expression levels of CD8 positive T cells within the tumor tissues (15, 16). The MSI-H CRC exhibits an active immune microenvironment probably due to recognizing many tumor neoantigens (17, 18). In this patient, the IHC assay showed a small number of CD8 positive T cells infiltrated within the tumor tissue, and many CD8 positive T cells were expressed in the tumor stroma, which may have contributed to the failure of immunotherapy.

The keynote-158 clinical trial has confirmed that TMB is a robust biomarker for predicting the efficacy of PD1 mab, with higher TMB indicating better efficacy (19). For TMB detection, peripheral blood and tumor tissues are generally selected. In this case, we found that the TMB in the peripheral blood was higher than in the tumor tissue. We speculated that a large amount of ctDNA in the lesion was released into the peripheral blood after the rapid tumor progression. In contrast, the tumor tissue only represented the TMB in this site.

Previous reports have suggested that MSI-H or immune-infiltrated tumors have evolved mutations that may confer resistance to recognition by the immune system in untreated samples (5). It is observed that *APC* biallelic mutations associate with increased WNT signaling and decreased TILs in MSS and MSI-H tumors (20). Cen et al. reported that mutant *APC* promotes tumor immune evasion *via* PD-L1 in CRC (21). In our case, we found that this patient had an *APC* mutation, which may lead to resistance to anti-PD1 therapy. However, the protein expression of PDL1 was negative in the tumor tissue of this patient, suggesting that in addition to *APC* gene mutation, there may be other gene dysfunction leading to immune tolerance. It is reported that *PTEN* gene mutation also correlated with response to anti-PD1 therapy. Chida K et al. showed that *PTEN* gene mutations in MSI-H/dMMR gastrointestinal tumors often did not respond to PD1 mab therapy (22). In addition, *KRAS* gene mutations are associated with poor anti-PD1 efficacy (23). In this patient, *PTEN* mutations were detected in the tumor tissue, and *KRAS* mutations were detected in the tumor tissue and peripheral blood. Therefore, the above gene mutations may be related to the failure of PD1 mab treatment. This suggests that the tumor immune microenvironment is very complex, and the factors determining immunotherapy’s efficacy still need further exploration. Therefore, the patient’s tumor microenvironment characteristics and detailed genomic status must be comprehensively evaluated before anti-PD1 mAb treatment, even for MSI-H tumors. In addition to MSI status, other gene mutations that may affect the therapeutic effect should also be considered comprehensively in screening the population with anti-PD1 treatment advantage among CRC patients.

In summary, this case may have significant clinical implications for MSI-H CRC patients’ resistance to anti-PD1 mab therapy, especially those with LS-related MSI-H CRC.

## Data availability statement

The original contributions presented in the study are included in the article/supplementary material. Further inquiries can be directed to the corresponding author.

## Ethics statement

Ethical review and approval was not required for the study involving human participants in accordance with the local legislation and institutional requirements. Written informed consent was obtained from the patient for the publication of any potentially identifiable images or data included in this article.

## Author contributions

XQ conceived the idea of the article. QZ composed the manuscript and figure-making. JH and LL supported the clinical data. TW and YZ provided the IHC test. All authors contributed to the article and approved the submitted version.

## Funding

This study was supported by Jiangsu Provincial Natural Science Foundation (SBK2021021543), the Nanjing Science and Technology Development Project (ZKX21028), and the High-level Innovation and Entrepreneurship Talent Introduction Plan of Jiangsu Province (JSSCBS20211532).

## Conflict of interest

The authors declare that the research was conducted in the absence of any commercial or financial relationships that could be construed as a potential conflict of interest.

## Publisher’s note

All claims expressed in this article are solely those of the authors and do not necessarily represent those of their affiliated organizations, or those of the publisher, the editors and the reviewers. Any product that may be evaluated in this article, or claim that may be made by its manufacturer, is not guaranteed or endorsed by the publisher.
